# Influence of Cultural Beliefs on the Utilization of Integrated Maternal, Newborn, and Child Health Services in Benue State, Nigeria

**DOI:** 10.7759/cureus.52808

**Published:** 2024-01-23

**Authors:** Chima C Igbokwe, James T Ihongo, Lawreta I Abugu, Cylia N Iweama, Jacinta E Ugbelu

**Affiliations:** 1 Department of Human Kinetics and Health Education, University of Nigeria, Nsukka, NGA; 2 Department of Community Health, NKST College of Health Technology, Mkar, NGA

**Keywords:** health service utilization, taboos, cultural norms, cultural beliefs, integrated maternal newborn and child health

## Abstract

Background

The maternal mortality ratio in Nigeria is high at 576 per 100,000 live births. The health policy is in favor of health advocacy programs that promote the utilization of integrated maternal newborn and child health (IMNCH) services in local primary health centers by childbearing mothers. Cultural factors, however, have been shown to limit the widespread adoption of these services. The purpose of this study was to investigate the influence of cultural beliefs on the utilization of IMNCH services by child-bearing mothers (CBMs) in Benue state.

Methods

A community-based correlational survey research was conducted on a random sample of 1,200 CBMs. A multistage sampling technique was used to select the study participants and a pretested, structured questionnaire was used to collect data through face-to-face interviews. Only 896 copies of the distributed Integrated Maternal Newborn and Child Health Services Utilization Questionnaire (IMNCHSUQ) had complete information and were used for analysis. The collected data were managed and analyzed using SPSS version 25 (IBM Corp., Armonk, NY). Mean, standard deviation, and point-biserial correlation were used to answer the research questions while linear regression was used to test the null hypotheses at a 0.05 level of significance.

Results

The majority of the CBMs were married (79.7%) and unemployed (66.0%) while the predominant age group was between 15 and 24 years (42.7%). CBMs had a high level of utilization of IMNCH services (X ®=3.30, SD=0.94); there was a positive moderate relationship between IMNCH utilization and cultural factors (*r_bp_*=.43, ρ= 0.000). Results also suggest that cultural factors were significant predictors of IMNCH services utilization in Benue State, Nigeria.

Conclusion

Any health education program for maternal and child health in Benue State should take cognizance of the cultural values, beliefs, and norms of the people to sustain positive ones while discouraging values and norms detrimental to the health and well-being of CBMs and their children.

## Introduction

Maternal and child health services have emerged as one of the most important public health interventions that determine global and national well-being. This is because every individual, family, or community is, at some point, intimately involved in pregnancy, the success of childbirth, and the post-partum period [1]. Although recent global estimates indicate a decline in maternal and child mortality, regional data show that it remains relatively high in low- and middle-income countries, accounting for 86% of reported maternal deaths worldwide [1,2]. Nigeria has the second highest estimated maternal deaths globally and the highest neonatal mortality deaths in Africa placing the lifetime risk of death from pregnancy, childbirth, or the postpartum period at 1 in 22 [3]. Several factors have been described around the adequacy of quality supply of maternal and child health care at different levels of care and the utilization of health services by women [4]. However, overall, the availability and utilization of Integrated Maternal, Newborn, and Child Health (IMNCH) services can lead to improved healthcare outcomes and bring the country closer to achieving the 2030 targets of the Sustainable Developmental Goals (SDGs) [5].

IMNCH services prepare childbearing mothers (CBMs) for positive outcomes during pregnancy, childbirth, and the postpartum period. By providing health education and increasing awareness about maternal health, the IMNCH service helps in the early detection of mothers at high risk of morbidity and mortality during the antenatal period and during postnatal care [6,7]. Despite the increased infrastructural and personnel development in Nigeria, the national average of maternal mortality remains one of the highest contributors to global prevalence with significant local state-to-state variations. Recent evidence has however shown that these improvements in infrastructure and personnel are still inadequate for supply and are also still under-utilized because of predominant cultural and economic factors [4,8,9].

Several reports have identified cultural factors as a limitation to the utilization of IMNCH services in Nigerian communities. Women in northern Nigeria have been reported to have more early marriages that expose young girls to early childbearing and may also prevent women from showing signs of distress from pregnancy [10]. Early childbearing has been associated with worse maternal outcomes and may lead to morbidities such as ectopic pregnancy, preterm labor, premature rupture of membranes, and vesicovaginal fistulae, and may lead to maternal death [10,11]. Low-income communities and lack of education constitute factors responsible for restricted access to information about antenatal care services and may lead to maternal morbidity and mortality. Perceived fear of contraceptive side effects deters the utilization of family planning services leading to the adoption of folkloric or traditional methods that are ineffective and expose women to unwanted pregnancies, unsafe abortions, and maternal mortality [8,12,13]. There are however variations in the utilization of IMNCH services from state to state and all these contribute to the country’s national average maternal mortality outcomes [4]. The dearth of literature on the utilization of IMNCH services in Benue State formed the backdrop for this study. Some cultural factors might limit the ability of CBMs to make decisions for their health and the health of their newborns and children, thereby exposing them to maternal, infant, and child morbidity and mortality. Hence, this study aimed to determine the level of utilization of IMNCH services and the relationship between cultural factors and utilization of IMNCH by CBMs in Benue State, Nigeria. The study also tested the null hypothesis of no significant relationship between cultural factors and the utilization of IMNCH services by CBMs in Benue State.

## Materials and methods

Study setting

The area of study is Benue State, Nigeria. It is in the North Central region of Nigeria. It is one of the 36 states of Nigeria with an approximate population of 5,741,800 according to the 2016 projected population [14]. The rural and urban LGAs have at least one functional healthcare facility. Some are private while others are government-owned health facilities where CBMs receive health care services.

Study design

A retrospective correlational survey research design was used.

Sample size determination and sampling procedure

A sample size of 1,200 childbearing mothers was determined using a proportion formula in line with the literature; they were selected and used for the study [15]. A 5% significance level and error margin were used, and an additional 10% of the minimum sample size was added to account for potential non-response or other sources of data loss.

The multistage sampling procedure was used for sample selection. In the first stage, two senatorial districts were selected out of the three senatorial districts in Benue State using a simple random sampling technique of balloting without replacement. Subsequently, three LGAs each were selected from the two selected senatorial districts using a simple random sampling technique of balloting without replacement. Thus, a total of six LGAs were used for the study. Forty-eight PHCs (eight from each LGA) were selected using purposive sampling. Furthermore, Federal Medical Centre (FMC), Makurdi, and Benue State University Teaching Hospital, Makurdi, were purposively selected for the study because of the high volume of IMNCH services they provide. In the fourth stage, convenience sampling was used to select 1,200 CBMs (24 from each facility) who gave consent to participate in the study. The women were selected at the health facilities during ANC visits and immunization days.

Data collection tool and analysis

The data collection instrument was the Integrated Maternal, Newborn, and Child Health Services Utilization Questionnaire (IMNCHSUQ) designed by the researchers. The IMNCHSUQ consisted of 18 items and was divided into three sections: A, B, and C. Section A consisted of seven items that elicited information on the demographic factors of the CBMs. Section B consisted of six items that elicited information on the level of utilization of IMNCH services among CBMs. Section C consisted of five items on cultural factors that influence the utilization of IMNCH services by women. Section B of the questionnaire was assigned on a 4-point Likert-type response option of always (4 points), sometimes (3 points), rarely (2 points), and never (1 point). The respondents were required to place a tick (√) against the items in the questionnaire that best described their level of utilization in the past 12 months. Section C consisted of five items on cultural factors that influence the utilization of IMNCH services by CBMs. Questions here include: cesarian section is evil; puerperal fever and psychosis after delivery are caused by evil spirits; blood transfusion is a taboo; health facilities should be visited during ANC, delivery, or when there is complication; and preferred place of delivery. The questions were designed to cover cultural norms, beliefs, taboos, and values. This section was assigned the dichotomous response options of “Yes” or “No”. The respondents were required to place a tick (√) against the factors they perceived to influence their use of IMNCH services.

The face validity of the IMNCHUSQ was established by giving a draft copy of the instrument, purpose of the study, research questions, and hypotheses to five experts from the researchers’ institution. The reliability index of the IMNCHSUQ was determined using Cronbach’s alpha and the split-half method for sections B and C, respectively. The reliability indexes of .97 and .84, respectively, were obtained for the entire scale. Thus, the instrument was considered reliable [15]. One thousand two hundred (1,200) copies of the questionnaire were administered by the researcher and five research assistants to the respondents in their respective health facilities. The literate CBMs filled the responses under the supervision of the researcher and research assistants. Research assistants interpreted the contents of the questionnaire in the local language to illiterate mothers.

The completed copies of the questionnaire were examined for completeness of responses. Copies of the questionnaire with incomplete information were not used for data analysis. The collected data were analyzed using the Statistical Package for Social Science (SPSS) Batch System Version 25 (IBM Corp., Armonk, NY, USA). Both descriptive and inferential statistics were employed in data analysis. Descriptive statistics such, as mean, standard deviations, and point-biserial correlation, were used to answer the research questions while inferential statistics, such as simple linear regression, were used to test the null hypotheses. To determine the extent of utilization of IMNCH services, the real limits of numbers were employed. This implies that mean (x) scores that range from 0.00 - 1.49 indicated a low level of utilization; mean scores from 1.50 - 2.49 indicated a moderate level of utilization; mean scores that ranged from 2.50 - 3.49 were considered a high level of utilization; and mean scores that ranged from 3.50 - 4.00 were interpreted as a very high level of utilization.

To interpret the direction and strength or magnitude of the relationships among the variables, Nwagu and Agbaje guidelines for the interpretation of correlation coefficients were used [16]. Thus, a correlation coefficient that ranges from ±.00 to ±.29 was interpreted as No to weak relationship (NR/WR); correlation coefficients that range from ±.30 to ±.59 was interpreted as moderate relationship (MR); and values from ±.60 to±.99 were interpreted as strong relationship (SR). Simple linear regression was used to test the null hypotheses at a 0.05 level of significance.

Ethical approval

Ethical clearance was obtained from the Ministry of Health and Human Services, Benue state, Nigeria (MOH/STA/204/VOL.1/39). The administrative offices in each facility were informed about the purpose of the study and permission was obtained. Informed consent was taken from study participants after informing the objectives and benefits of the study. All information was kept confidential.

## Results

Socio-demographic characteristics of respondents

Table 1 shows the characteristics of the women who participated in the present study (n=896). Most of the participants were married (n=714, 79.7%) and unemployed (n=591, 66.0%). The predominant age group was between 15 and 24 years (n=383, 42.7%). The educational level of all the respondents was a minimum of primary education while 55.5% (n=497) completed tertiary education. There was a near-even spread of the respondents, as 58.9% (n=528) of the respondents were dwellers of urban areas (Table 1).

**Table 1:** Sociodemographic characteristics of respondents

S/N	Characteristic	Frequency	Percentage (%)
1.	Place of residence		
	Rural area	368	41.1
	Urban area	528	58.9
2.	Age group (years)		
	15-24	383	42.7
	25-34	243	27.1
	35-44	259	28.9
	45 and above	11	1.2
3.	Parity		
	1-2 children	369	41.2
	3-4 children	392	43.6
	5 children and above	135	15.1
4.	Marital status		
	Single	6	0.7
	Married	714	79.7
	Divorced	152	17.0
	Widowed	16	1.8
	Separated	8	0.9
5.	Educational level		
	Primary education	50	5.6
	Secondary education	349	39.0
	Tertiary education	497	55.5
6.	Religion		
	Christianity	864	96.4
	Islam	26	2.9
	African Traditional Religion (ATR)	6	0.7
7.	Employment status		
	Employed	305	34.0
	Unemployed	591	66.0

Table 2 shows that overall, childbearing mothers highly utilized IMNCH services ( X= 3.30, SD = 0.94). A very high level of utilization was identified for tetanus vaccination (X = 3.50 SD = 0.84), delivery in the hospital by skilled birth attendants (X = 3.53 SD = 0.85), and neonatal care services such as the regular provision of immunization (X = 3.56 SD = 0.87) and sleeping under insecticide-treated nets (X = 3.55 SD = 0.85).

**Table 2:** Level of utilization of IMNCH services by CBMs (n = 896) x = mean, SD = standard deviation X, 0.00 – 1.49 = Low level of utilization (LLU); X, 1.50 – 2.49 = Moderate level of utilization (MLU); X, 2.50 – 3.49 = High level of utilization (HLU); X, 3.50 – 4.00 = Very high level of utilization (VHLU); IMNCH: integrated maternal newborn and child health; CBM: child-bearing mother

Items	X	SD	Decision
Family planning (in the last 12 months)			
Screening services for diseases before your first pregnancy	3.09	0.99	HLU
Health education services for the prevention of unwanted pregnancy/birth spacing	3.41	0.89	HLU
Cluster	3.25	0.94	HLU
Antenatal care services			
Tetanus vaccination	3.50	0.84	VHLU
Treatment of malaria (i.e., intermittent treatment for prevention of malaria)	3.49	0.82	HLU
Nutritional education for improvement of fetal/child and maternal nutrition	3.38	0.78	HLU
Prevention of communicable diseases	3.18	0.99	HLU
Blood test for anemia, diabetes, hypertension, or HIV	3.24	1.09	HLU
Abdominal examination	3.31	0.92	HLU
Measurement of weight and height for prevention of gestational overweight/obesity	3.29	0.98	HLU
Cluster	3.34	0.92	HLU
Delivery care services			
Abdominal scan (ultrasonography) and vaginal examination	3.34	0.96	HLU
Delivery in the hospital by skilled birth attendants (i.e., nurses, midwives, doctors)	3.53	0.85	VHLU
Examination of placenta for completeness	3.41	0.97	HLU
Use of sterilized equipment, such as scissors/scalpel, by the birth attendant during delivery	3.41	0.92	HLU
Palpation of the abdomen for stimulation and detection of the baby’s position	3.27	0.95	HLU
Cluster	3.39	0.93	HLU
Post-natal care services			
Checking of vital signs and general condition after delivery	3.40	0.94	HLU
Immunization services such as tetanus toxoid.	3.14	1.03	HLU
Health education on food preparation and weaning	3.28	0.99	HLU
Counseling services for family planning during postnatal visit	3.14	0.95	HLU
Visited place of delivery for immunization/check-up within 6 weeks of delivery	3.04	0.93	HLU
Advise on personal and environmental hygiene.	3.20	0.92	HLU
Cluster	3.20	0.96	HLU
Newborn care services			
Regular provision of immunization (i.e., routine and supplemental immunization)	3.56	0.87	VHLU
Consumption of an adequate diet for the promotion of exclusive breastfeeding	3.37	0.97	HLU
Instruction on the need for adequate rest and sleep	3.23	1.02	HLU
Sleeping under an insecticide-treated net (ITN)	3.55	0.85	VHLU
Use of skilled newborn care services	3.27	0.92	HLU
Cluster	3.39	0.93	HLU
Child healthcare services			
Regular clinic checkup/screening	3.24	0.92	HLU
Child’s growth monitoring	3.09	1.01	HLU
Health education on childcare practices for mothers.	3.18	1.00	HLU
Demonstration of oral rehydration therapy (ORT) for the prevention of diarrhea	3.25	0.91	HLU
Health education on the treatment of minor illnesses such as fever and cough	3.26	0.92	HLU
Nutrition education for mothers on exclusive breastfeeding (EBF)	3.40	0.86	HLU
Cluster	3.24	0.94	HLU
Grand mean	3.30	0.94	HLU

Table 3 shows that overall, there is a positive moderate relationship between IMNCH utilization and cultural factors (*r_bp_* = 0.43, ρ = 0.00). Specifically, there are positive moderate relationships between the utilization of family planning (*r_bp_* = 0.33, ρ = 0.00), antenatal care services (*r_bp_* = 0.39, ρ = 0.00), delivery care service (*r_bp_* = 0.44, ρ = 0.00), postnatal care service (*r_bp_* = 0.43, ρ = 0.00), and child health care service (*r_bp_* = 0.45, ρ = 0.00) and cultural factors. A strong relationship was found between newborn care services (*r_bp_* = 0.55, ρ = 0.00) and cultural factors. This implies that cultural factors influence the utilization of IMNCH services among CBMs.

**Table 3:** Point biserial correlation between the utilization of IMNCH and cultural factors ±0.00 - ±0.29 = No relationship to weak relationship (WR); ±0.30 -±0.59 = Moderate relationship (MR); ±0.60 -±0.99 = Strong relationship (SR). *r_bp_* = point biserial correlation; IMNCH: integrated maternal newborn and child health

Items	Cultural factors		
	r_bp_	P-val.	Decision
Family planning			
Screening services for diseases before your first pregnancy	0.15	0.00	WR
Health education services for the prevention of unwanted pregnancy/birth spacing	0.41	0.00	MR
Cluster	0.33	0.00	MR
Antenatal care services			
Tetanus vaccination	0.43	0.00	MR
Treatment of malaria (i.e., intermittent treatment for prevention of malaria)	0.41	0.00	MR
Nutritional education for improvement of fetal/child and maternal nutrition	0.43	0.00	MR
Prevention of communicable diseases	0.19	0.00	WR
Blood test for anemia, diabetes, hypertension, or HIV	0.17	0.00	WR
Abdominal examination	0.31	0.00	MR
Measurement of weight and height for prevention of gestational overweight/obesity	0.24	0.00	WR
Cluster	0.39	0.00	MR
Delivery care services			
Abdominal scan (ultrasonography) and vaginal examination	0.29	0.00	WR
Delivery in the hospital by skilled birth attendants (i.e., nurses, midwives, doctors)	0.42	0.00	MR
Examination of placenta for completeness	0.43	0.00	MR
Use of sterilized equipment such as scissors/scalpel by birth attendant during delivery	0.44	0.00	MR
Palpation of the abdomen for stimulation and detection of the baby’s position	0.27	0.00	WR
Cluster	0.44	0.00	MR
Postnatal care services			
Checking of vital signs and general condition after delivery	0.35	0.00	MR
Immunization services such as tetanus toxoid	0.29	0.00	WR
Health education on food preparation and weaning	0.25	0.00	WR
Counseling services for family planning during postnatal visit	0.28	0.00	WR
Visited place of delivery for immunization/check-up within six weeks of delivery	0.23	0.00	WR
Advise on personal and environmental hygiene.	0.30	0.00	MR
Cluster	0.43	0.00	MR
Newborn care services			
Regular provision of immunization (i.e., routine and supplemental immunization)	0.44	0.00	MR
Consumption of an adequate diet for the promotion of exclusive breastfeeding	0.48	0.00	MR
Instruction on the need for adequate rest and sleep	0.38	0.00	MR
Sleeping under an insecticide-treated net (ITN)	0.44	0.00	MR
Use of skilled newborn care services	0.29	0.00	WR
Cluster	0.55	0.00	SR
Child healthcare services			
Regular clinic checkup/screening	0.37	0.00	MR
Child’s growth monitoring	0.27	0.00	WR
Health education on childcare practices for mothers.	0.21	0.00	WR
Demonstration of oral rehydration therapy (ORT) for the prevention of diarrhea	0.39	0.00	MR
Health education on the treatment of minor illnesses such as fever and cough	0.45	0.00	MR
Nutrition education for mothers on exclusive breastfeeding (EBF)	0.41	0.00	MR
Cluster	0.45	0.00	MR
Overall cluster	0.43	0.00	MR

Table 4 shows the result of simple linear regression conducted to assess the relationship between the utilization of IMNCH services and cultural factors. The results show that there was a significant relationship between cultural factors and the utilization of family planning services (β= 0.377, < 0.05), antenatal care services (β= 1.399, < 0.05), delivery services (β= 1.160, < 0.05), post-natal care services (β= 1.175, < 0.05), and newborn care services (β= 1.403, < 0.05). The R^2^ value implies that cultural factors explained the percentage of the variance in the utilization of IMNCH by CBMs in the model. Therefore, the R^2^ value of 0.11 implies that cultural factors explained 11% of the variance in the family planning services utilization by CBMs in the model.

**Table 4:** Summary of simple linear regression of the relationship between the utilization of IMNCH services and cultural factors of CBMs (n = 896) Dependent variables – utilization of family planning services, antenatal care services, delivery care services, post-natal care services, newborn care services, child health care services Independent variable = cultural factors; ρ < 0.05; β = unstandardized coefficient; IMNCH: integrated maternal newborn and child health; CBM: child-bearing mother

Variables	Utilization of IMNCH			
		95% CI for β		
	β	Lower bound	Upper bound	p-value
Family planning services				
Constant	2.996			0.000
Cultural factors	0.377	0.305		0.000
R	0.33			
R^2^	0.11			
Antenatal care services				
Constant	10.391	8.358	12.423	0.000
Cultural factors	1.399	1.182	1.616	0.000
R	0.39			
R^2^	0.15			
Delivery care services				
Constant	6.215	4.774	7.655	0.000
Cultural factors	1.160	1.006	1.314	0.000
R	0.44			
R^2^	0.19			
Post-natal care services				
Constant	8.320	6.806	9.834	0.000
Cultural factors	1.175	1.014	1.337	0.000
R	0.43			
R^2^	0.19			
Newborn care services				
Constant	4.329	3.055	5.604	0.000
Cultural factors	1.365	1.229	1.501	0.000
R	0.55			
R^2^	0.30			
Child healthcare services				
Constant	6.399	4.674	8.124	0.000
Cultural factors	1.403	1.219	1.587	0.000
R	0.45			
R^2^	0.20			

## Discussion

Level of utilization of IMNCH services

Findings in Table 2 showed that childbearing mothers had a high level of utilization of IMNCH services (X=3.30, SD=0.94). This can be attributed to the various successful maternal health interventions by government and non-government agencies that have been implemented in these facilities in a bid to reduce the high MMR of Benue state [17,18]. Recent reports have shown that the maternal mortality ratio (MMR) in Benue State is as high as 1189 per 100,000 population, making the state one of the highest contributors to the national average of 576 deaths per 100,000 [17,19]. As a result of this, several strategies have been implemented by government and non-government agencies (including Meta) to contain the menace [17,18,20]. The utilization of IMNCH services in Nigeria is also largely affected by the cost of care [9,21,22]. Government agencies and development partners have contributed to a heavily subsidized cost of antenatal and postnatal care in Nigeria. They have also invested in health campaigns in the region and, in some cases, offered cash rewards for the utilization of IMNCH through the Subsidy Reinvestment and Empowerment Program (SURE-P), which increased the utilization of delivery services [23,24].

A cross-sectional analysis of maternal healthcare utilization in sub-Saharan Africa also showed that young women in sub-Saharan Africa utilize delivery services much more than antenatal services [25]. This study presents a similar finding, revealing that delivery services (X = 3.53 SD = 0.85) had very high utilization. Additionally, women who decided against antenatal care must have chosen to utilize the delivery services due to the increased symptomatology and difficulty associated with labor. The cost of regular ANC visits and the distance of the health care facility could also pose challenges to reduced ANC utilization in Benue state [9,26]. ANC utilization remains an essential component of IMNCH and should be encouraged for early identification of high-risk pregnancies and also because it encourages increased utilization of postnatal services [13,25]. Therefore efforts should be directed to increasing antenatal care utilization. Although the utilization of IMNCH services is acceptable, efforts are necessary to scale up the availability of these services to improve reach and further reduce the rate of maternal mortality in Benue state.

Relationship between utilization of IMNCH services and cultural factors

This study showed a positive moderate relationship between IMNCH services utilization and cultural factors (r_bp_= 0.43, ρ= 0.00). This implies that cultural factors influence the utilization of IMNCH services among CBMs. The high utilization of IMNCH services by CBMs, as shown in Table 2, does not connote optimal utilization. Careful observation of findings shows that the level of utilization was affected by traces of cultural factors, thereby revealing significant relationships in the level of utilization of IMNCH services. A linear regression of this result showed significant variance (Table 4) attributable to cultural factors in the levels of utilization of the IMNCH services, and this has been shown in similar reports.

Traditional and religious beliefs have been highlighted as key determinants of health-seeking behavior for CBMs in previous studies going as far as forbidding skilled pregnancy care [10,25,27]. In a traditional system, wives are also dependent on their husbands for the cost of accessing care and this also impacts the access to care because the husband must be convinced of the necessity, especially in the ‘absence’ of a symptom [27,28]. This buttresses the established association between early marriage, early childbearing (<24 years), and adverse pregnancy outcomes [29,30]. Therefore a combination of these factors in Benue State aggravates the indices of maternal mortality in the state. For this reason, delaying pregnancy, educating women, and community-based strategies to implement family planning are key considerations to reduce maternal mortality [29]. The preference for large family sizes among husbands also exposes women to a higher risk of maternal deaths with previous high-risk pregnancies [28]. These beliefs and practices introduce significant barriers to antenatal care services, and family planning services and limit the optimal overall utilization of IMNCH services [13,29]. Thus, education and empowerment of CBMs are key instruments to better position them for optimal utilization of IMNCH services. Health educators and professionals must raise awareness through strategic programs to encourage women on the benefits of IMNCH services.

One major limitation of the study is the use of a self-reported questionnaire. The respondents could have been biased in their responses to impress the researcher. The use of a mixed-method approach would have given more robust results. However, the findings are an eye-opener to the influence of cultural factors on IMNCH utilization in the study area, which can be leveraged for future studies. Future research is essential in determining the burden of maternal and newborn morbidity and mortality attributable to cultural factors. Although we have reviewed the cultural relationship of IMNCH services as a cluster, further research may be essential to assess the relationship of cultural factors with specific IMNCH components and to suggest service improvements in line with cultural perspectives.

## Conclusions

In conclusion, there was a very high level of utilization of delivery services in Benue State. Other IMNCH services in Benue State also showed high utilization; however, IMNCH service utilization is predicted by the influence of early childbearing, unemployment, large family sizes, and dependence on male spouses, which leads to suboptimal utilization of these services and the consequent adverse outcomes. Education and empowerment of CBMs will increase women's status through labor force participation, alleviate the burden of poverty and unemployment, and ease the delivery of educational materials that favor the utilization of IMNCH.
